# Small-Scale Dynamic Aurora

**DOI:** 10.1007/s11214-021-00796-w

**Published:** 2021-02-01

**Authors:** Ryuho Kataoka, Christopher C. Chaston, David Knudsen, Kristina A. Lynch, Robert L. Lysak, Yan Song, Robert Rankin, Kiyoka Murase, Takeshi Sakanoi, Joshua Semeter, Tomo-Hiko Watanabe, Daniel Whiter

**Affiliations:** 1 National Institute of Polar Research, 10-3 Midori-cho, Tachikawa, Tokyo, 185-0031 Japan; 2 Space Sciences Laboratory, University of California, Berkeley, CA 94720 USA; 3 Dept of Physics and Astronomy, University of Calgary, Calgary, AB T2N 1N4 Canada; 4 Dept of Physics and Astronomy, Dartmouth College, Hanover, NH 03755 USA; 5 School of Physics and Astronomy, University of Minnesota, Minneapolis, MN USA; 6 SOKENDAI, 10-3 Midori-cho, Tachikawa, Tokyo, 185-0031 Japan; 7 Planetary Plasma and Atmospheric Research Center, Aramaki-aza-Aoba 6-3, Aoba, Sendai, Miyagi 980-8578 Japan; 8 Department of Electrical and Computer Engineering and Center for Space Physics, Boston University, Boston, MA USA; 9 Department of Physics, Nagoya University, Nagoya, 464-8602 Japan; 10 Physics & Astronomy, University of Southampton, SO17 1BJ Southampton, UK

**Keywords:** Magnetosphere-ionosphere coupled region, Auroral phenomena, Auroral breakup, Flickering aurora, Dispersive Alfven waves, Ionospheric feedback instabilities, Ionospheric Alfven resonator

## Abstract

Small-scale dynamic auroras have spatial scales of a few km or less, and temporal scales of a few seconds or less, which visualize the complex interplay among charged particles, Alfvén waves, and plasma instabilities working in the magnetosphere-ionosphere coupled regions. We summarize the observed properties of flickering auroras, vortex motions, and filamentary structures. We also summarize the development of fundamental theories, such as dispersive Alfvén waves (DAWs), plasma instabilities in the auroral acceleration region, ionospheric feedback instabilities (IFI), and the ionospheric Alfvén resonator (IAR).

**Supplementary Information:**

The online version contains supplementary material available at 10.1007/s11214-021-00796-w.

## Introduction

For the purposes of this review, we define small-scale, dynamic aurora to include optical and plasma structure having characteristic spatial scales of a few km or less, and time scales of a few seconds or less. On these scales, spatial and temporal effects are often tightly intertwined. The justification of these choices is as follows.

Until the 1960’s, scientific study of auroral morphology depended on eyewitness accounts and drawings, and later, film-based cameras having limited resolution in space and time, as exposure times of many tens of seconds were required due to the relatively low light levels of most auroral phenomena (Stormer 1955). Despite these limitations, many key types of auroral phenomena were categorized and given names such as luminous bands, curtains, omega bands, pillars (Vallance-Jones 1974). One of the most common and recognizable forms apparent in conventional film-based photographs of the period is now known as a stable auroral arc, which often dominates the evening-time and pre-midnight auroral sky, is highly elongated in the east-west direction, and has characteristic north-south widths of the order of 10’s of km (Kim and Volkman 1963). Using a sounding rocket, McIlwain (1960) established that such arcs are due to magnetic field-aligned electron beams having energies of several keV. Later these were termed “inverted-V” (also “discrete”) arcs due to their signatures in energy-time spectrograms recorded by polar-orbiting satellites (Frank and Ackerson 1971). Early satellite observations based on spinning photometers on ISIS-II also established a broad region of unstructured emission (at least down to the resolution of those composite images) at the equatorward edge of the auroral zone, termed the “diffuse” aurora (Lui and Anger 1973).

Through naked-eye observations, it was known in those early days that the aurora contains features which are more highly structured in space (scales of ∼1 km and smaller) and time (several seconds or less) than could be recorded with conventional film cameras, for example flickering aurora, which oscillates at frequencies of the order of 10 Hz (Beach et al. 1968). In a landmark paper, Maggs and Davis (1968) used an image-intensified TV camera with a telescopic lens to record fine structure within the aurora having characteristic widths as small as 70 m. Borovsky and Suszcynsky (1993) confirmed the existence of these small-scale features, and Borovsky (1993) pointed out that, at the time, no auroral theories could account for them. A reanalysis of the Maggs and Davis (1968) observations by Stenbaek-Nielsen et al. (1999) concluded that those particular fine structures were in fact not associated with discrete arcs, but instead were embedded within the diffuse aurora. More recent observations (Dahlgren et al. 2012) have since shown that even discrete arcs may contain fine structure down to scales of 0.09 km.

The renewed interest in small-scale auroral structures following Borovsky and Suszcynsky (1993) led some to view ∼100 m as the “true” scale of the aurora, and that mesoscale arcs were in fact assemblages of such small structure. However, Knudsen et al. (2001) demonstrated that in the case of mesoscale arcs observed by more modern, CCD-based all-sky cameras, north-south structure of stable arcs is dominated by Gaussian-like profiles with a characteristic widths of 10-30 km, and with little sub-structure down to the camera resolution of 1.7 km. In other words, small-scale (km or sub-km scale) aurora is distinct from quiet, mesoscale phenomena such as discrete arcs.

As will be described below, rapid advancements in camera technology in the past two to three decades have led to extensive observations of small-scale and dynamic auroral features, and have established that small-scale aurora is also a common feature of auroral breakup following substorm onset.

The spatiotemporal properties of small-scale auroras were reviewed in Sandahl et al. (2008) and Sandahl et al. (2011). After the successful ground-based observations utilizing EMCCD cameras (e.g., Semeter et al. 2008) and sCMOS cameras (e.g., Dahlgren et al. 2013), new types of small-scale auroras have also been found by these fine resolution observations. The ASK project was the most successful in revealing the energy flux associated with fine structures using three identical EMCCD cameras with multiple wavelengths. For example, Dahlgren et al. (2008) reported that “filaments” or “curls” were the energetic part of surrounding auroras. Dahlgren et al. (2011) reported that thin arcs are related to increase in number flux without an increase in energy.

**Fig. 1:**
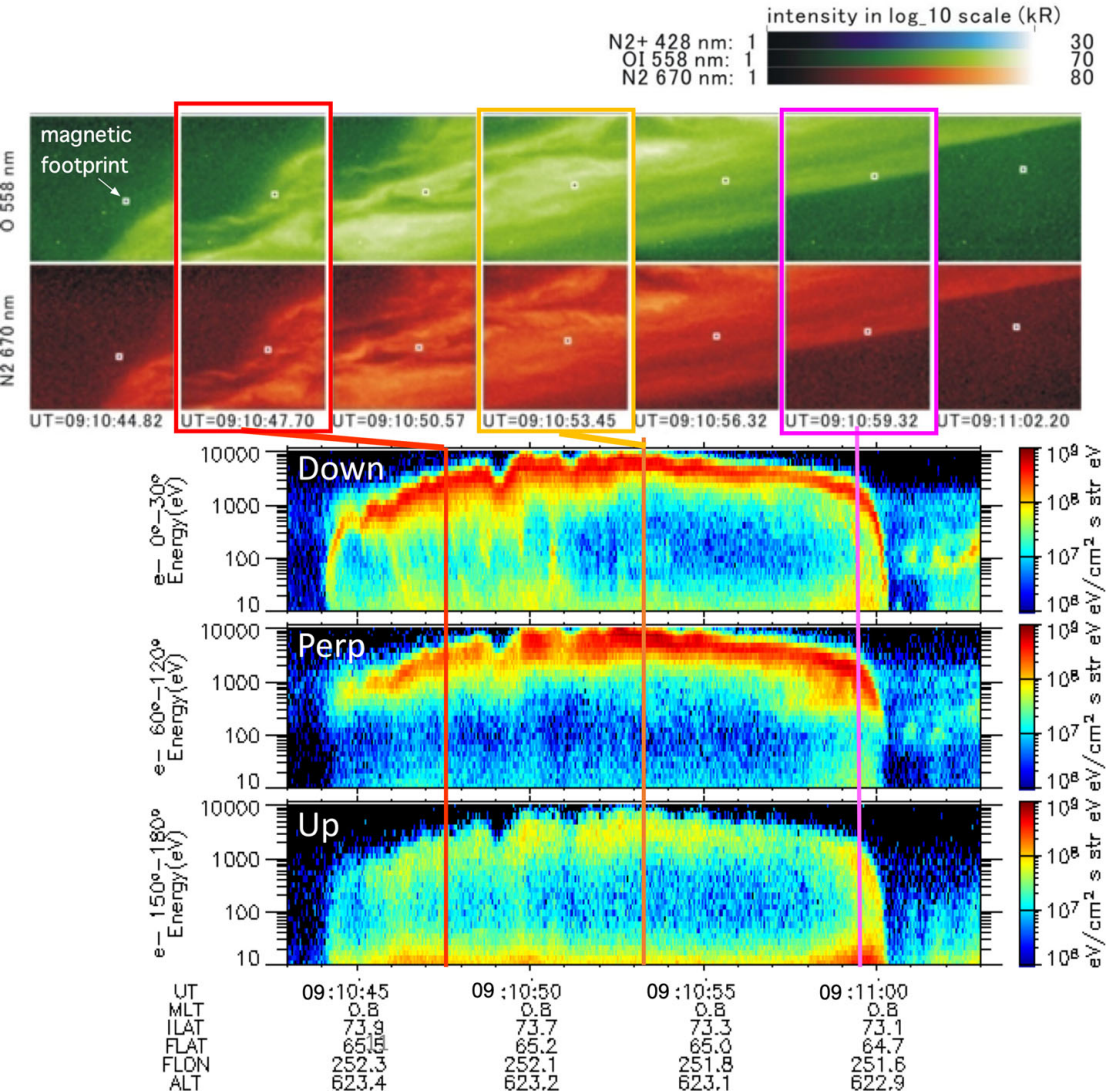
Inverted-V electrons and auroral arcs observed by the Reimei satellite on 2005-12-26, moving across the auroral band structure with a latitudinal width of about 100 km. Multiple small-scale auroral arcs showed sheared motions as shown in Movie 1

A breakthrough was achieved by the Reimei satellite observations. The Reimei satellite has capabilities of simultaneous auroral imaging and auroral electron measurement with a spatial resolution of ∼1 x 1 km and a time resolution of 120 ms (Asamura et al. 2003; Sakanoi et al. 2003), and revealed the precise relationship between auroral arcs and precipitating electrons (Asamura et al. 2009; Frey et al. 2010, Chaston et al. 2010, 2011, Fukuda et al. 2014, Motoba and Hirahara 2016). Figure 1 shows an event of inverted-V electrons and auroral arcs observed by Reimei. In this case, Reimei observed auroral band structure with a latitudinal width of about 100 km. Note that there are multiple small-scale auroral arcs showing sheared motions (Fig. 1, see also Movie 1) in the poleward half of the auroral band, and rather uniform emission in the equatorward part, which is consistent with the previous finding that there is often a band-like emission of several tens of km in the background when a small-scale auroral arc appears (Haerendel 1999). In fact, electron data show the inverted-V electron precipitation with widths of ∼100 km corresponding to the auroral band structure. In the poleward half of the inverted-V structure where the small-scale auroral arcs appeared, there are small-scale fluctuations of the peak energies of electrons in the energy range of several keV, with time-dispersed low-energy electrons precipitating simultaneously, implying the existence of Alfvén wave acceleration (Whiter et al. 2012). It was concluded from this event that the small-scale auroral arcs are not generated by a single acceleration mechanism but produced by the fluctuations of peak energy of inverted-V type accelerated electrons as caused by the interaction of Alfvén waves.

As another example of the important role of Alfvén waves in the auroral acceleration region, Asamura et al. (2009) suggested that Alfvén waves are generated by the shear instability (Wu and Seyler 2003) using the Reimei data over auroral arcs with fast shear flows (Movie 2). In this case, Alfvén waves are a secondary process, generated by the shear flow in the magnetosphere-ionosphere coupled region.

Many researchers have considered that inertial Alfvén waves play an important role in auroral acceleration in the several thousands of km altitudes where a low-$\beta $ condition is satisfied. When the wavelength of obliquely propagating inertial Alfvén waves $\lambda _{\bot }$ becomes comparable to the electron inertial length $\lambda _{e}$, a field-aligned acceleration electric field is formed (e.g., Stasiewicz et al. 2000a,b). Chaston et al. (2003b) estimated that the auroral width generated from inertial Alfvén waves can be 0.5-1.0 km. However, within this range, the acceleration energy is about 0.1-0.5 keV, which is an order of magnitude smaller than auroral precipitating electron energy that produces typical auroral arcs. Chen et al. (2005) demonstrated that Alfvén waves can produce modulation of inverted-V accelerated electrons in the keV energy range. Their previous studies suggested that the small-scale auroral structures are produced by coupling of multiple acceleration mechanisms including inertial Alfvén waves rather than a single acceleration mechanism. The vast majority of our discussion below will therefore be related to the important role of Alfvén waves in causing small-scale dynamic auroras.

In a recent study using suprathermal (100-350 eV) electron measurements on ePOP in addition to field measurements from Swarm, Wu et al. (2020b) carried out a statistical study of periodic electron bursts which recurred several times at periods consistent with the eigenfrequency of the ionospheric Alfvén resonator (IAR). These events are similar to those reported by Chaston et al. (2002a, 2002b) using the FAST satellite in the nightside auroral region, and Tanaka et al. (2005) using a sounding rocket in the dayside cusp, but extend the number of events to more than 50, and show that they are distributed over a wide range of magnetic local times (from pre-noon to post-midnight) and geomagnetic latitudes (64-78 degrees). In at least one case, the electron bursts observed by ePOP were associated with auroral rays, a connection reported previously by Lynch et al. (2012) based on sounding rocket observations, and indicating a possible coupling between rays and the IAR. Wu et al. (2020b) were also able to reproduce detailed properties of electron energy and pitch-angle dispersion observed within individual bursts by tracing test particles within a downward-propagating Alfvén wave.

Other sounding rocket observations have also contributed to the understanding of the IAR and ionospheric feedback. The Auroral Current and Electrodynamics Structure (ACES) mission observed small-scale electric and magnetic perturbations together with measurements of precipitating electrons to investigate the properties of the IAR (Cohen et al. 2013). They found results consistent with the ionospheric feedback model of Streltsov and Lotko (2008), including the structuring of the auroral currents and formation of a low density cavity. Studies of the IAR and feedback were also a focus of the Magnetosphere-Ionosphere Coupling in the Alfvén Resonator (MICA) sounding rocket program (Lynch et al. 2015). They compared their results with a simple electrostatic model based on both the in situ field measurements, and on the PFISR radar observations of the ionosphere (Zettergren et al. 2014), and showed that the model could not reproduce the small-scale structures, which they then interpreted as possibly being caused by feedback interactions in the IAR.

In Sect. 2, we review specific phenomena representing the small-scale dynamic aurora, such as vortices, filaments, and packets. We can classify the majority of the phenomena as a variety of localized Alfvénic interactions on multiple scales and morphologies. However, it is an open question as to how we can model the Alfvénic electron acceleration associated with the rapid formation and deformation of double layers, plasma instability, and turbulent cascading. In Sect. 3 we discuss recent developments in the essential theories, such as dispersive Alfvén waves, feedback instability, and the IAR. In Sect. 3.4, concluding remarks of this review article are summarized.

## Specific Phenomena

### Flickering

Bright auroras sometimes show localized flickering intensity modulation (Movies 3 and 4). The modulation frequency is typically 3-15 Hz and the typical size of the bright patch is 1-12 km across magnetic field lines at an emission altitude of 100 km (Kunitake and Oguchi 1984; Sakanoi and Fukunishi 2004; Michell et al. 2012). The modulation frequency is consistent with the O^+^ ion-cyclotron frequency in the auroral acceleration region (Temerin et al. 1986; Lund et al. 1995) and the spatial structure is consistent with an interference pattern associated with dispersive Alfvén waves (Sakanoi et al. 2005; Gustavsson et al. 2008; Kataoka et al. 2011b; Yaegashi et al. 2011). Whiter et al. (2008) showed that flickering intensity increases when background non-flickering aurora becomes brighter.

Ground-based high-speed observations provide an important clue for understanding the possible generation mechanism. Whiter et al. (2010) identified “chirps” (variation in frequency over 1-2 s) in multi-spectral optical observations of flickering aurora, and found that the electron precipitation energy has an inverse correlation with the flickering frequency during these chirps, consistent with electron acceleration by Landau resonance with dispersive Alfvén waves. The leading edge of moving flickering patches was shown by Kataoka et al. (2011b) to be more energetic than the trailing edge, which could be a result of dispersive Alfvén waves losing energy by Landau damping. Recent advances in ground-based imaging techniques such as EMCCD cameras have allowed us to find zenith-view flickering auroras with frequencies above the oxygen ion-cyclotron frequency of auroral acceleration regions (Yaegashi et al. 2011). Using an sCMOS camera Fukuda et al. (2017) found 60-80 Hz flickering aurora, which can be modulated by proton-band electromagnetic ion cyclotron (EMIC) waves, coincident with 10 Hz flickering and therefore indicating the presence of multi-ion EMIC waves. The fastest-varying patch was smaller than the 10 Hz patch. The fastest variation of side-view flickering aurora was 180 Hz as observed with a photometer (McHarg et al. 1998).

Related phenomena include dispersive electrons called field-aligned bursts (FABs) as observed by sounding rockets above the ionosphere (Andersson et al. 2002), and EMIC and broad-band ELF (BBELF) waves as observed by satellites in the magnetosphere-ionosphere coupled regions (Erlandson et al. 1994, McFadden et al. 1987; Lund et al. 1998). The flickering auroras therefore manifest complex interactions among electrons and ions, and dispersive Alfvén waves nearby auroral acceleration regions.

### Vortices

**Fig. 2:**
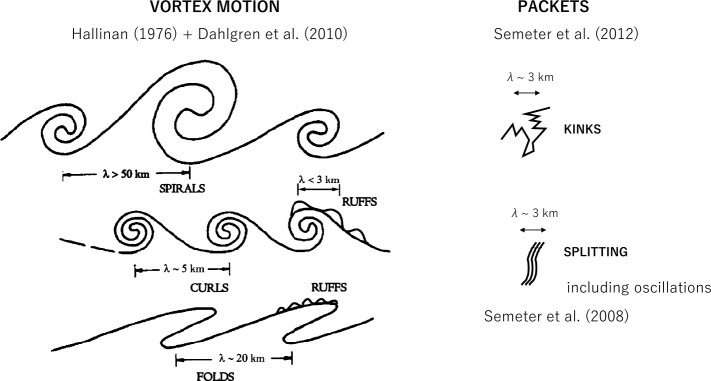
Cartoon of the magnetic zenith-view of small scale auroral structures. (After Hallinan 1976; Dahlgren et al. 2010). The packet structures are compared against vortex motion (Semeter et al. 2008; Semeter 2012)

Km-scale vortex-like structures of the aurora are known as “curls” (Hallinan 1976; Trondsen and Cogger 1998; Vogt et al. 1999) (Fig. 2, see also Movies 5 and 6). More recently, smaller-scale structure was found along curls with inverse rotation and named “ruffs” (Dahlgren et al. 2010) (Fig. 2, see also Movie 7). Ivchenko et al. (2005) found that “curls” were caused by precipitation of energetic electrons without a low-energy population, while both high and low energy precipitation were present in the “rays.” Another well-known small-scale vortex-like structure is “folds”, which have typical scale of ∼10 km (Fig. 2, see also Movie 8). Recently, the formation and inverse cascade of folds were found just before auroral breakup (Kataoka et al. 2011a), which is similar to so-called auroral “beads” appearing at substorm onset (Motoba et al. 2012). Wavy structures which look different from typical folds were also reported at the poleward boundary of multiple arcs, along with eddies and flickering (Kataoka et al. 2015).

These small-scale vortex structures in the magnetic zenith view have long been considered as the cause of “rays” appearing in side-viewed auroral curtains. However, a different type of side-view appearance of “rays” was also found at poleward boundary intensification (PBI) by Lynch et al. (2012), where sounding rocket observations showed electrons of ionospheric origin accelerated by Alfvén waves. Sample images of these PBI rays are shown in Movie 9.

### Filaments

Progressive advances in imaging technologies applied to auroral research have led to fundamental advances in our understanding of the connections between auroral morphology and the associated energy source. The goal of understanding “filamentary structure” in the aurora has arisen as a distinct quest in auroral physics, based partly on this technological progression. The lower minimum spatial scale perpendicular to the ambient magnetic field appears to be 70∼90 m (Maggs and Davis 1968; Borovsky 1995; Dahlgren et al. 2012). This limit corresponds to the electron inertial length near the density minimum in the near-Earth magnetosphere, suggesting that inertial Alfvén wave dispersion plays an important role in producing these structures (e.g., Stasiewicz et al. 2000a, 2000b).

Bulk properties of the incident particle flux can be deduced from the auroral spectrum in the magnetic field-aligned direction, and recent observational programs have sought to bring such a multi-spectral imaging capability to auroral research (Dahlgren et al. 2008; Ivchenko et al. 2005; Grubbs et al. 2018). One notable result has been the discovery of spectral signatures consistent with higher energy (>8 keV) mono-energetic particle fluxes within filamentary features observed in the magnetic zenith (Dahlgren et al. 2012). This population was subsequently found to be co-mingled on the same flux tube with a higher energy ∼30 keV population (Dahlgren et al. 2015). This result is consistent with findings of Arnoldy et al. (1999) who suggested that broad-band field-aligned bursts may be correlated with modulation of the entire inverted-V potential drop. Such co-mingling has also been recently observed for filament structure embedded within the diffuse aurora (Sivadas et al. 2019), and may also explain observation of flickering observed in sub-kilometer features (Whiter et al. 2008). These results do not call into question the importance of Alfvén waves in modulating the aurora, but they suggest an interplay among energization mechanisms powering the aurora that may not be fully understood.

**Fig. 3:**
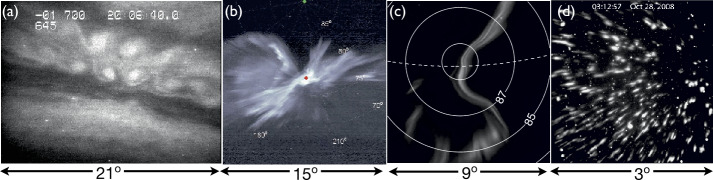
Ground-observations of fine scale auroral phenomena with fields-of-view as shown: **a**) Auroral curls (Vogt et al. 1999), **b**) “Flaming” auroral filaments (Dahlgren et al. 2013), **c**) Auroral “packets” (Semeter et al. 2008), **d**) Decameter filaments produced by HAARP (Kendall et al. 2010)

Ionospheric heating experiments have been shown to create filamentary aurora-like emissions with spatiotemporal characteristics similar to natural auroras (Kendall et al. 2010) (Fig. 3, see also Movie 10). Furthermore, for heater facilities co-located near an ISR facility, these features are found to be accompanied by enhanced ion-acoustic backscatter, similar to NEIALs (Kosch et al. 2007). These results of heater experiments such as HAARP and EISCAT HF facility have contributed substantially to our understanding of small-scale M-I coupling (Streltsov et al. 2018) and have guided simulation studies (Akbari et al. 2016).

### Packets

An auroral breakup is synonymous with the impulsive release of magnetic stress in the magnetosphere-ionosphere system. The resultant energy influx leads to creation and modulation of field and particle fluxes embodying a rich variety of spatial and temporal scales (e.g., Kataoka, 2011). Although auroral displays are highly variable during these events, coherent patterns can nonetheless be identified in observations of sufficient resolution. One intriguing and repeatable phenomenon is the bifurcation of narrow arcs into systems of parallel forms (Trondsen et al. 1997; Semeter and Blixt 2006; Semeter et al. 2008). This dynamic is often superposed on a moving reference frame, giving the appearance of a translating wave packet (Fig. 3, see attached Movie 11). Semeter et al. (2008) have termed such motion “dispersive” (as opposed to “fluid”), in that there is a distinctly different phase velocity (motion of individual arc elements) and group-velocity (motion of the packet) in the observer frame of reference. The packet formation is qualitatively consistent with the auroral projection of an Alfvén resonant cone (Singh 1999).

Such patterns in auroral breakup are not confined to arc-like features. Dahlgren et al. (2013) observed periodic cylindrical filaments racing upward along the magnetic field. Termed “flaming aurora,” a careful analysis of these signatures using a high frame-rate sCMOS sensor revealed consistency with dispersive field-aligned bursts (FABs) commonly observed by particle detectors in regions of Alfvénic turbulence (Lynch et al. 1994). The appearance of these Alfvénic relationships between these features and the particle source populations remains poorly understood. Borovsky (1995) once concluded that “*Existing auroral-arc theories do not explain the finescale structures because none of these theories will allow a structure as narrow as the observed finescale structures*”. Indeed, the thesis put forth by Borovsky (1995) appears to still hold.

### Effects on the Ionospheric State

The fields and precipitation patterns associated with small-scale auroral phenomena produce small-scale irregularities in the ionosphere, affecting radar backscatter, trans-ionospheric radio propagation, and the global-scale M-I energy budget. Electron distribution functions associated with filamentary aurora are spread broadly in energy, extending from several keV’s down to thermal levels, as discussed in Sect. 3. The low-energy portion can interact resonantly with Langmuir and ion-acoustic modes in the ionosphere (Akbari et al. 2012). Such interactions are detected by Incoherent Scatter Radar (ISR) as “Naturally Enhanced Ion Acoustic Line” (NEIAL) (Grydeland et al. 2003). Coordinated observations with optical sensors have been used to correlate NEIAL features with specific types of auroral structure (Blixt et al. 2005; Michell and Samara 2010; Akbari et al. 2012). The region of destabilization presents a source of anomalous resistivity, which has been implicated in regulating macro-scale M-I processes such as ion outflow (Forme and Fontaine 1999).

Irregularities induced by small-scale auroral processes also affect ground-space communication links and Global Navigation Satellite Systems (GNSS). Recent work has found GPS signal disruptions, in the form of scintillation and complete loss-of-lock, to be clustered at the trailing edge of an auroral surge, suggesting a structuring mechanism involving the interplay between precipitation-induced gradients, and field-induced drift (i.e., gradient drift instability) (Semeter et al. 2017). Such technological effects of fine-scale auroral dynamics may have important implications for space weather forecasting in newly opened arctic shipping routes.

## Theories and Discussions

### Dispersive Alfvén Waves and Field-Aligned Electron Acceleration

**Fig. 4:**
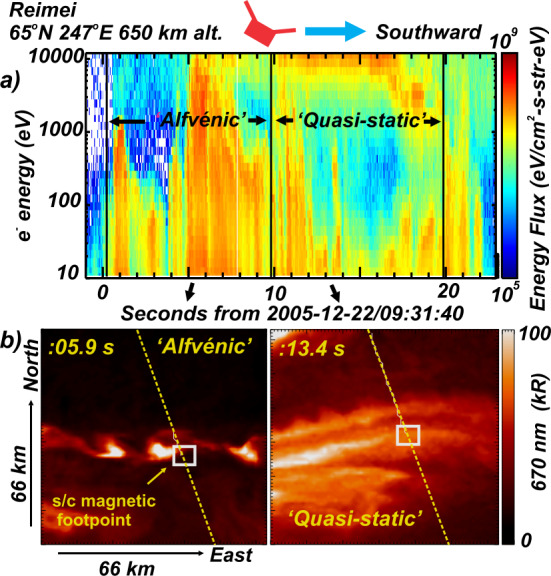
Auroral electron and magnetically conjugate camera observations of rapidly evolving auroral forms returned from the Remei spacecraft. (**a**) Electron energy spectrogram revealing the electron energy fluxes responsible for auroral emission at 670 nm shown for (**b**) Alfvénic and (**c**) Quasi-static aurora (After Chaston et al. 2010)

While specific auroral forms are distinguished by their morphology and evolution, in-situ measurements of the electron acceleration processes are distinguished by the form of the electron spectrum (Paschmann et al. 2003). Figure 4 presents measurements returned from the Reimei spacecraft showing electron spectra (a) and conjunctive imagery (b and c). On the left the electron spectrum is broad and characteristic of what is often termed the ‘Alfvénic aurora’ while on the right there is a distinct peak at ∼10 keV representing what is termed an ‘inverted-V’ (Lyons 1981). The latter is often identified as the ‘quasi-static’ aurora because the electron spectrum is largely invariant over many Alfvén bounce times between the acceleration region and ionosphere. While the physics of Alfvén waves is central to the dynamics and structure of both the auroral forms shown in Figs. 2b and 2c in Chaston et al. (2010), in the case of ‘Alfvénic aurora’ dispersive Alfvén waves are responsible for the electron acceleration itself. In fact, the correlation of broad-spectrum electromagnetic field fluctuations in the ELF range with bursts of broad spectrum field-aligned as shown in Fig. 4a electrons is an established feature of the in-situ observations above the auroral oval (Andersson et al. 2002; Chaston et al. 2003a; Lynch et al. 1996; Knudsen et al. 1998). Measurements show that the relative variation of the electric and magnetic spectra of these fluctuations with spacecraft frame frequency are in general consistent with the properties of a broad $k$-spectrum of dispersive Alfvén waves (Wahlund et al. 1998, 2003; Stasiewicz et al. [Bibr CR176][Bibr CR177]; Chaston et al. 1999, 2008; Hull et al. 2010, 2016). Dispersive Alfvén waves have wavelengths transverse to the background magnetic field of the order of characteristic plasma scales including the electron inertial length ($\lambda _{e} = {c} / {\omega _{pe}} $), ion gyro-radius ($\rho _{i} = {\left ( T_{i} / m_{i} \right )^{{1} / {2}}} / {\Omega _{i}}$) and ion acoustic gyro-radius ($\rho _{s} = {\left ( T_{e} / m_{i} \right )^{{1} / {2}}} / {\Omega _{i}} $). Here $\omega _{pe}$ is the electron plasma frequency, $\Omega _{i}$ is the ion cyclotron frequency, $T_{i}$ is the ion temperature, $T_{e}$ is the electron temperature, and $m_{i}$ the ion mass. Unlike MHD Alfvén waves the propagation of these modes through the plasma requires the existence of a significant wave electric field component parallel to the background magnetic field ($E_{\parallel } $) (Lysak and Lotko 1996). It is this property that is responsible for the field-aligned electron distributions commonly observed within these waves. Detailed reviews of the properties of these waves and the acceleration processes that operate within them can be found in Stasiewicz et al. (2000b), Chaston (2006), Birn et al. (2012) and Mottez (2014).

Field-aligned electron bursts observed within dispersive Alfvén waves above the auroral oval are typically impulsive, broad in energy (‘broadband’ or supra-thermal bursts), or time dispersed features on sub-second timescales extending from at most a few keV down to the eV range (Andersson et al. 2002). These features can occur in isolation or in combination with classical inverted-V type electron spectra (e.g. Kletzing and Hu 2001; Chaston et al. 2002a; Asamura et al. 2009). Efforts to explain the characteristics of these features have in general relied on a linear fluid description of the perpendicular field and plasma dynamics coupled to the electron momentum equation (Thompson and Lysak 1996) or Vlasov equation (Watt and Rankin 2012) along the geomagnetic field. The relationship between the parallel electric field and the plasma can be most easily understood by considering the force balance described by the electron momentum equation where,

1 $$ E_{\parallel} = \mu _{o} \lambda _{e}^{2} ( \frac{\partial J_{\parallel}}{\partial t} + v_{\bot } \cdot \nabla _{\bot } J_{\parallel} + J_{\parallel} \nabla _{\parallel} \frac{J_{\parallel}}{q_{e} n_{e}} )- \frac{\nabla _{\parallel} P_{e\mid \mid }}{q_{e} n_{e}} - \frac{(P_{e\mid \mid } - P_{e\bot } ) \nabla _{\parallel} B_{o} / B_{o}}{q_{e} n_{e}}. $$

Here $J_{\parallel} = n_{e} q_{e} v_{\parallel } $ is the field-aligned current, $P_{e}$ is the electron pressure, $v_{\bot } $ and $v_{\parallel } $ are velocities perpendicular and parallel to the geomagnetic field, $q_{e}$ is the electron charge, $n_{e}$ is the electron density and $B_{o}$ is the geomagnetic field strength. For cold electrons and small-scale waves at low altitudes (≲2 R_E_ above the surface) where $\lambda _{e} > \rho _{s}$ the first term on the RHS of Eq. (1) dominates and the waves are generally termed inertial Alfvén waves (Goertz and Boswell 1979; Lysak and Carlson 1981; Stasiewicz et al. 2000a,b). At higher altitudes $\rho _{s} > \lambda _{e}$ the second term becomes more important and the waves are known as kinetic Alfvén waves (Hasegawa 1976; Lysak and Lotko 1996). The third term describes the parallel electric field balancing the mirror force acting on primarily hot plasma sheet current carrying electrons. For the zero-frequency case the integrated contribution of this term along the geomagnetic field corresponds to the well-known Knight relation (Knight 1973). For time varying fields this contribution has been evaluated analytically using Vlasov or gyrokinetic approaches (Rankin et al. 1999a,b; Nakamura 2000; Lysak and Song 2003; Watanabe 2014) and via numerical simulation (Watt 2010; Damiano and Johnson 2013). For the highly field-aligned distributions characteristic of electron bursts in dispersive Alfvén waves this contribution is likely not the dominant driver of field aligned electron bursts. Consequently, efforts to model the electron acceleration mechanism leading to these bursts have largely focused on the inertial and electron pressure gradient contributions (Chaston et al. 2003a, 2003b, 2003c). Conjunctive observations from spacecraft separated along the geomagnetic field and via imaging suggest that these effects drive field-aligned electron acceleration over many Earth radii along auroral field-lines (Wygant et al. 2000, 2002; Keiling et al. 2002; Chaston et al. 2005; Dombeck et al. 2005; Damiano et al. 2018)

Test particle simulations have been widely performed to understand the generation of field-aligned electron bursts in model wavefields propagating along realistic altitude dependent phase speed profiles. These have shown how the macro-scale features of the geomagnetic field and plasma distribution together with the micro-physics of the wave-particle interaction conspire to define the observed form and evolution of the electron distribution in velocity space (Thompson and Lysak 1996; Chaston et al. 2000, 2002a, 2002b; Kletzing and Hu 2001; Andersson et al. 2002; Su et al. 2004; Chen et al. 2005). How this works can be qualitatively understood from a consideration of the field-aligned potential ($\phi $) in the wavefield, the electron velocity ($v_{\parallel } $) and the parallel wave phase speed ($\frac{\omega }{k_{\parallel }} $) expressed as,

2 $$ \frac{\omega }{k_{\parallel }} - \sqrt{\frac{2 q_{e} \phi }{m_{e}}} < v_{\parallel } < \frac{\omega }{k_{\parallel }}. $$

Here $\omega $ is the wave frequency, $k_{\parallel } $ the wavenumber along $B_{0}$ and $m_{e}$ is the electron mass. Any electron with $v_{\parallel } $ in this range can be picked up by the wave and carried along with speed of the order of $\frac{\omega }{k_{\parallel }} $. The fact that the geomagnetic field varies as the inverse cube of the radial distance from the Earth, while the mass density of the plasma is relatively constant above a few thousand kilometers altitude, means that an Alfvén wave propagating from a magnetospheric source will speed-up as it approaches Earth. This provides an accelerator for electrons which become resonant with the wave according to Eq. (2) at high altitudes where the Alfvén speed is less than the electron thermal speed and the resonant location in phase space occurs where significant phase space densities are found. Consequently, large fractions of the source electron distribution may become resonant with the wave. These resonant electrons are then driven Earthward with progressively increasing speed as the phase speed of the wave increases (Chaston 2006; Watt and Rankin 2009). The convergence of the geomagnetic field over this path focusses both the wave energy density and electron flux to account for appreciable energy deposition (Keiling et al. 2002, 2003, 2019; Chaston et al. 2003a, 2007a,b; Hatch et al. 2016, 2017, 2018; Dombeck et al. 2018)

The broad flat-top like distributions that this interaction generates are a consequence of the differences in energization due to stochastic interaction with different wave phases, trapping (Clark and Seyler 1999; Su et al. 2004; Watt and Rankin 2009; Damiano et al. 2015), and variations in energy gain depending on the source altitude of the accelerated an electron (Chaston et al. 2000, 2002a; Andersson et al. 2002). The special case of time dependent energy dispersed bursts observed at low altitudes as part of this process occurs specifically through the action of large amplitude impulsive coherent field variations. The source altitude that the dispersion infers corresponds to the upper edge of the topside ionosphere where the acceleration process terminates due to increasing electron densities (Kletzing and Hu 2001; Chaston et al. 2002b).

While test particle simulations provide basic insight into the manner in which the acceleration occurs, the fact that the field-aligned potential in the wavefield is often comparable to the temperature of the supporting plasma means that a self-consistent non-linear approach is required. In a series of papers Watt et al. (2004, 2005, 2006, 2009), Watt and Rankin (2010, 2012) using a Vlasov model have shown how the bulk of the electron population remains non-resonant with the wave and carries the field-aligned wave current while a fraction are resonantly accelerated to energies of the order of twice the Alfvén speed. It has been suggested that these two processes naturally provide a dispersive burst terminated by a non-dispersive ‘broadband’ or supra-thermal burst as the wave passes over the spacecraft. Features having this form are commonly observed (Andersson et al. 2002). Hybrid fluid-kinetic approaches have also been implemented (Hui and Seyler 1992; Clark and Seyler 1999; Swift 2007a,b; Damiano et al. 2015, 2016). The results from Damiano et al. have for example explored the dependency of the electron energization on the relative ion to electron temperature ratio and finite ion gyro-radius effects along the high-altitude portion of an auroral field-line. This work along with that by Swift (2007a) has revealed the importance of wave dispersion across the geomagnetic field as a constraint on the energization process. This dispersion is largest in the presence of hot ions where finite gyro-radii effects spread wave energy across the geomagnetic field and can significantly reduce the effectiveness of the acceleration process. This suggests that the Alfvén wave acceleration process will be most effective on auroral field-lines connected to the cold dense plasma sheet and is consistent with the observation of the most energetic field-aligned electron bursts occur preferentially at high latitudes.

These simulation results provide basic physical understanding of why field-aligned electron distributions are observed in association with dispersive Alfvén waves. However further efforts are required to more credibly connect these processes to visible aurora and to incorporate the action of small-scale kinetic processes observed within the Alfvén wavefield. Imaging from the Reimei spacecraft show that the form of aurora driven by dispersive Alfvén waves are not consistent with the simplified 1-D or even 2-D model geometries assumed. Filaments and vortices are characteristic of Alfvénic aurora (Chaston et al. 2010). These features require a 3-D model and represent the action of non-linear instabilities in the perpendicular plane (Seyler 1990). While the parallel dynamics described above still apply it is likely that the mechanisms through which the acceleration is energetically supported is not just through field aligned Poynting flux at a particular wave scale as modelled. Seyler and Liu (2007) instead have performed 3-D particle in cell simulations that capture this morphology while also examining small scale kinetic interactions associated with wave breaking. This process is found to drive field-aligned electron acceleration while simultaneously driving ion acoustic waves. The later may be related to observations of large amplitude impulsive parallel electric fields in dispersive Alfvén waves (Stasiewicz et al. 1997; Chust et al. 1998; Chaston et al. 1999, 2007a,b; Ergun et al. 2005) suggestive of the formation of double layers (Lysak and Hudson 1987; Silberstein and Otani 1994) or more generally Debye scale regions of charge separation within larger scale Alfvén wave fields. Genot et al. (2004) have explored the coupling of the Alfvén wave to small scale kinetics to demonstrate how the focusing of wave energy into density cavities leads to the formation of phase space holes. These features have been observationally linked to the generation of supra-thermal electron bursts (Chaston et al. 2006). These PIC simulations that allow an examination of the detailed kinetics in the wave are necessarily performed on small scales so how they couple with, and perhaps modify, the larger scale parallel acceleration dynamics along the geomagnetic field remains undetermined.

### Dynamics of Small Scale Structured Aurora

Dispersive Alfvén waves and the electron acceleration discussed in the previous section propose a possible source of electron precipitation in regard to small scale structures of auroras. In addition to the particle source, one demands a physics mechanism elucidating structure formation and dynamics of small-scale auroras. The dynamics and structuring of small-scale features in auroral forms is in broad terms determined by the ionospheric response to current closure and ionization, phase mixing and non-linear evolution of Alfvén waves, and instabilities acting on auroral current sheets and potential structures through the acceleration region. For the general description of those processes that lead to curls, folds and filamentation, the strength of the geomagnetic field ($B_{0}$) along an auroral fluxtube conveniently allows the relevant physics to be formulated in terms of the dynamics perpendicular and parallel to $B_{0}$ (Seyler 1988; Song and Lysak 2006). The former can be approximated by the single fluid (ion) momentum equation describing force balance in the perpendicular plane and the latter by the parallel electron momentum equation (Eq. (1)) describing the self-consistent distribution of parallel electric fields along auroral flux tubes that drive field-aligned electron acceleration to form aurorae. The occurrence of double layers along auroral field-lines (Ergun et al. 2004) tells us that in principle the parallel dynamics should be characterized using a fully kinetic model, however achieving this in 3-D while faithfully incorporating the macroscale variation in plasma parameters extending several Earth radii from the auroral ionosphere remains computationally challenging. Solutions to these equations are constrained by boundary conditions prescribed by current closure through the ionosphere (Seyler 1990; Watanabe 2010), and driven at the high-altitude end by applied current and voltage profiles representing magnetospheric drivers often referred to as ‘generators’ (Lysak 1990). The momentum equation describing the perpendicular dynamics (e.g. Biskamp 2003; Eq. 2.7) can be expressed as

3 $$ \rho \left ( \frac{\partial v_{\bot }}{\partial t} + v_{\bot } \cdot \nabla _{\bot } v_{\bot } \right ) \approx J\times B- \nabla _{\bot } P\approx J_{\parallel} \times b_{\bot } + \frac{B_{o}}{\mu _{o}} \frac{\partial b_{\bot }}{\partial z}, $$

where $\rho $ is the mass density, $J$ is the total current, $b_{\bot } $ is the perturbed magnetic field,and $P$ is the plasma pressure. The very low values for plasma $\beta $ through the auroral acceleration region allow the contribution from $\nabla _{\bot } P$ to be ignored. Advection of structure with $v_{\bot } $ through the acceleration region (Seyler 1990) suggest that the dynamics described by this equation are projected onto upper atmosphere by precipitating electrons (Hallinan 1981). Consequently, the observed motion and structuring of auroral forms qualitatively reflect that of auroral acceleration structures. Rotational vorticity in auroral luminosity therefore represents that of $v_{\bot } $ through the acceleration region requiring that the convective non-linearity ($\rho v_{\bot } \cdot \nabla _{\bot } v_{\bot } $) on the LHS side on Eq. (3) be non-zero. On the other hand, the concentration of $J_{\parallel} $ into filaments suggests that the magnetic non-linearity ($J_{\parallel} \times b_{\bot } $) on the RHS of Eq. (3) is significant. For an ‘Alfvénic’ arc with $\omega \sim k_{\parallel } v_{A}$, where $v_{A}$ is the Alfvén speed, and $v_{\bot } \sim b_{\bot } v_{A} / B_{o}$, then Ampere’s law provides the ratio of the non-linear terms to the linear terms in Eq. (3) as,

4 $$ \sim k_{\bot } b_{\bot } / k_{\parallel } B_{0}. $$

This relationship shows that as an auroral arc becomes narrower ($\sim k_{\bot } b_{\bot }$ increases) so too does the importance of the non-linear force terms. If $\omega $ is defined by an Alfvén transit time to the ionosphere from the acceleration region at ∼1 Earth radii, then using models for the Alfvén speed profiles above the aurorae (e.g. Stasiewicz et al. 2000a,b) the dynamics of kilometer scale arcs with $J_{\parallel} \gtrsim 1\ \upmu \text{A}/\text{m}^{2}$ will be dominated by the non-linearities leading to instabilities (Seyler 1988). This recognition explains why small scale auroral features are usually observed to be distorted from laminar form.

**Fig. 5:**
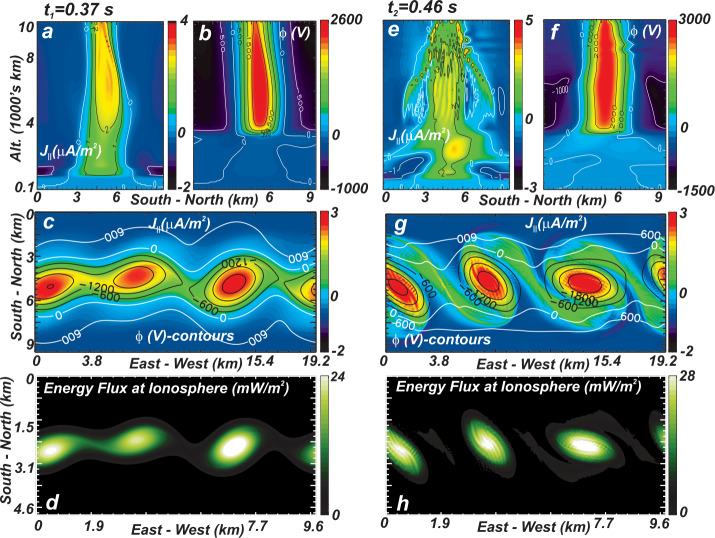
Snapshots in the evolution of an auroral current sheet for plasma parameters that provide M_sub_A > 1 and auroral ‘curls’ (After Chaston and Seki 2010). **a**) and **e**) show vertical structure, **c**) and **d**) show horizontal slices of the current and potential through the acceleration region while **g**) and **h**) show energy flux at the ionosphere. Spatial scales vary with altitude

**Fig. 6:**
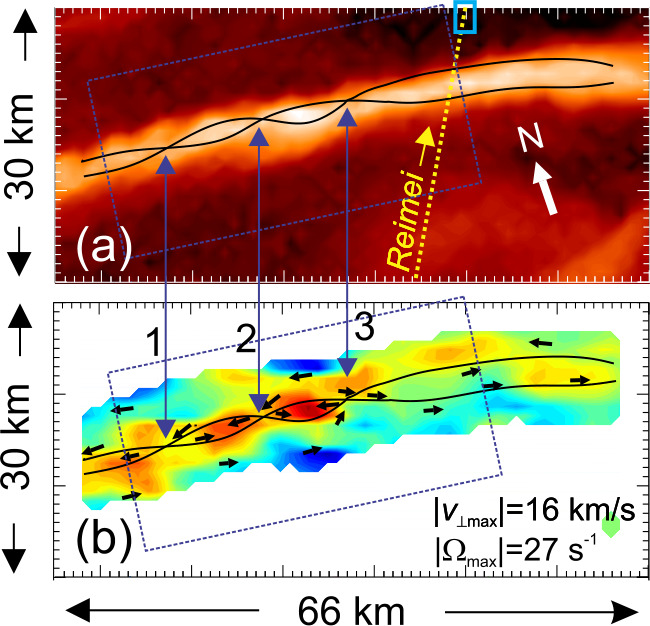
Observations of likely tearing instability in a discrete feature from the Reimei spacecraft (After Chaston 2015a,b). **a**) shows intensity at 670 nm while **b**) shows the corresponding optical vorticity with arrows indicating the direction of optical flow

A consideration of the relative magnitudes of the convective and magnetic non-linearities in Eq. (3) provides further insight into what drives the distortion of auroral forms in the auroral acceleration region. The ratio of these terms reduces to the Alfvén Mach number $M_{A} = \left ( \Delta v/\Delta v_{A\bot } \right )$ where $\Delta v_{A\bot } = \Delta b_{\bot } / \sqrt{\mu _{o} \rho } $, and $\Delta v$ and $\Delta b_{\bot } $ are respectively the change in velocity and magnetic field across the arc (Chaston and Seki 2010). Values greater than one may give rise to instabilities driven by flow shear (e.g. Kelvin Helmholtz; Fig. 5) thought to lead to the features described above as ‘folds’ and ‘curls’ (Hallinan and Davis 1970) while values less than one lead to instabilities driven by magnetic shear (e.g. tearing, Seyler 1990) identified in vortices observed from the Reimei spacecraft (Chaston 2015a,b; Fig. 6). For Alfvénic arcs on kilometer scales electron inertial effects in the acceleration region provide $M_{A} >1$ favoring flow shear. This local picture is however complicated by reflections from the conducting ionosphere, and/or density gradients at the base of the acceleration region that alter the relationships between the field and flows along the flux tube (Lysak and Dum 1983; Seyler 1988). Alternatively, for ‘quasi-static’ arcs, as described by the current voltage relation $J_{\parallel } =-K\phi$, we find $M_{A} =1/ \mu _{o} \Delta x^{2} v_{A} K$ where $\Delta x$ is the arc width and $K$ is the field-line conductivity defined for example by Knights relation (Knight 1973). In this model the cross-over between magnetic-shear or flow-shear dominance for kilometer scale features and acceleration region values for $v_{A}$ occurs at $K\sim 10^{-9}$ (ohm m^2^)^−1^. It is interesting to note that $K\sim 10^{-9} $ (ohm m^2^)^−1^ is a typical value derived from in-situ observations above discrete aurora (Lyons 1981; Sakanoi et al. 1995; Elphic et al. 1998). This suggests that both flow shear and magnetic shear are intrinsic to the observed evolution of discrete auroral forms. Moreover, the dependence on $K$ is indicative of the importance of the electron kinetics in defining the evolution of auroral arcs and from a macroscopic perspective represents of the importance of the properties of the electron source region that in turn define $K$. Qualitatively, hot tenuous magnetospheric source regions will tend to drive the evolution through flow shears while cooler more dense source region plasmas will provide evolutionary sequences that are more likely controlled by magnetic shear.

A detailed exposition of various instabilities active along an auroral flux-tube that can provide small scale structuring of auroral forms is provided in Seyler and Wu (2001) and Wu and Seyler (2003). Other relevant works not already cited include Wagner et al. (1983); Lotko et al. (1987); Chmyrev et al. (1992), Otto and Birk (1993), Rankin et al. (1993), Lysak and Song (1996), Shukla and Stenflo (1999), Peñano and Ganguli (2000) and Chaston et al. (2011) among others. How the processes described in these works, which operate largely through the acceleration region connected to a passive ionosphere, couple to an active and self-consistently evolving ionosphere is required for understanding the evolution of auroral displays on time-scales longer than an Alfvén bounce time. In the following section the representative case of coupling ionospheric feedback to the Kelvin-Helmholtz instability which is active through the acceleration region is described.

The feedback instability in the magnetosphere-ionosphere (M-I) coupling was first proposed as a mechanism of self-excitation of quiet auroral arcs as briefly summarized in the review of discrete arcs (Borovsky et al. 2020), and has been developed with extensions introducing a variety of physics models.

In the feedback M-I coupling model, the shear Alfvén waves propagating along auroral field lines carry the field aligned current $\tilde{j}_{\parallel } $ and the plasma motion $\tilde{v}$ given by the $E\times B$ drift, where tilde ($\tilde{\phantom{a}}$) denotes a perturbed quantity. Continuity of $\tilde{j}_{\parallel } $ and $\tilde{E}$ between the magnetosphere and the ionosphere constructs the M-I coupling, and induces auroral structuring with the ionospheric density change $\tilde{n}$. In the original feedback instability theory, the typical time scale ($\tau $) of auroras is characterized by periods of low-order Alfvén harmonics of field line resonance (say, order of a minute), and the horizontal scale at the ionospheric altitude is given by $V_{d} \tau $, where $V_{d}$ means the Pedersen or Hall drift speed in the auroral region. If $V_{d} \sim 100$ m/sec and $\tau \sim 100$ sec, one finds a simple estimate of the perpendicular (horizontal) wave length of fluctuations $\lambda \sim 10$ km which is comparable to latitudinal scales of auroral arcs. The feedback instability spontaneously generates auroral structures of $\tilde{j}_{\parallel } $, $\tilde{B}$, $\tilde{E}$, $\tilde{v}$, and $\tilde{n}$ in the form of a two-dimensional (obliquely propagating) plane wave, where $\tilde{B}$ and $\tilde{E}$ mean the perturbed electromagnetic fields with the same space-time scales as $\tilde{j}_{\parallel } $, $\tilde{v}$, and $\tilde{n}$. In the following, we discuss possible mechanisms or extensions of the feedback instability, leading to smaller-scale structures of auroras.

Now, let us consider extensions of the above model in a framework of the linear instability. In case with inhomogeneity of the Alfvén speed along the auroral field line, such as the IAR discussed below, a shorter time scale for the feedback instability is brought by the shear (possibly dispersive) Alfvén waves with higher frequencies (of the order of $\tau \sim 1$ sec; see the following subsection). It leads to excitation of auroral structures with smaller horizontal spatial scales of $\lambda \sim 100$ m, if we take into account the feedback resulting from M-I coupling with the IAR (Lysak 1991). It is remarked that the horizontal scale of auroras considered here stems from the field-aligned inhomogeneity of the Alfvén speed, and not from the perpendicular one resulting from phase mixing and wave propagation.

Extensions of the MHD equations with effects of the finite gyroradius and the electron inertia are essential to incorporate the parallel electric field $\tilde{E}_{\parallel } $ of the dispersive Alfvén waves and particle acceleration, and simultaneously introduce additional spatial scales such as the ion acoustic gyroradius or the electron skin depth. The small scales related to kinetic dynamics of magnetospheric plasma often lead to stabilization of short wavelength components and a cut off wavenumber for the feedback instability as found in the M-I coupling analysis based on the gyrokinetic theory (Watanabe 2014).

Introduction of pressure perturbations and field line curvature provides an additional instability in the M-I coupling. The ballooning instability in the MHD or kinetic regimes has widely been investigated as a possible mechanism for auroral beading (or wavy) structures. Since the cut-off wavenumber of the ballooning instability is given by extended MHD effects such as the finite ion gyroradius, small-scale structures can be generated even in the linear instability. An integrated modelling of the feedback and ballooning instabilities is recently developed by means of the reduced MHD framework of the three-dimensional M-I coupling (Watanabe 2020).

**Fig. 7:**
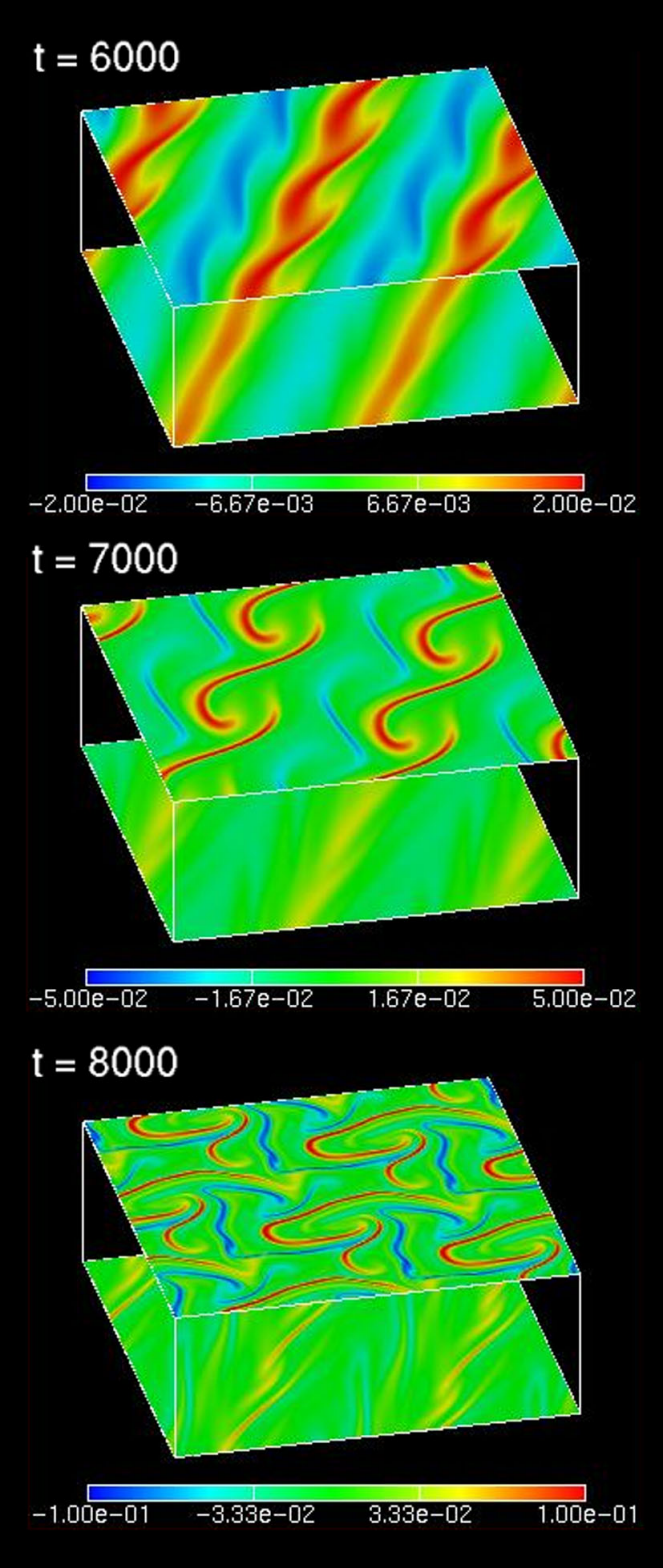
Color contours of the vorticity distributions on the ionosphere (lower plane) and the magnetic equator (upper plane) at three different time steps of the nonlinear simulation, where the vertical scale is shortened just for clarity of the plots. (After Watanabe 2010)

Nonlinear interactions of shear (or dispersive) Alfvén waves bring other possible mechanisms to create auroral fine structures through the feedback M-I coupling. Let us recall the feedback instability generates $\tilde{v}$, that is, the $E\times B$ flow driven by the perturbed electric field and the static magnetic field. Since Alfvén waves are accompanied by $\tilde{v}$, a horizontal sheared flow with a spatial scale of $\lambda $ is induced in the ionosphere. As the feedback instability grows, the flow speed as well as the velocity shear is amplified. If the flow shear exceeds a critical value, the Kelvin-Helmholtz (K-H) type instability is secondarily is switched on as shown in Fig. 7, if the nonlinear wave interaction is properly included in the M-I coupling (Watanabe 2010 and Watanabe et al. 2016). This is because the K-H instability is driven by the Reynolds stress coming from the advection term $\tilde{v} \cdot \nabla \tilde{v}$ in the equation of motion of a fluid. Here, the K-H instability secondary induced by the feedback instability leads to deformation of the primary arc structure, and generates vortex structures in the M-I coupling system, of which pattern has a similarity to folds observed in auroral breakup arc (Kataoka et al. 2011a,b). It is noteworthy to mention that the primary arc structure causing the K-H instability is also a result of spontaneous growth of the feedback instability, and is not *a priori* given by the initial set up of the system. Direct numerical simulation using the three-dimensional nonlinear M-I coupling model clearly captures the secondary K-H instability growth and transition to turbulence (Watanabe et al. 2016).

If the nonlinear interaction of the Alfvén waves propagating in the upward and downward directions is strong enough, the feedback instability develops into a fully turbulent state via the K-H instability growth. Interactions of counter propagating shear (or kinetic) Alfvén waves have widely been investigated in studies of the MHD turbulence (Goldreich and Sridhar 1995). In the quasi two-dimensional MHD turbulence with a strong external magnetic field, fluctuations with higher perpendicular wavenumbers are produced through interactions of the counter propagating shear Alfvén waves, leading to the energy cascade and the power spectrum of $k_{\bot }^{-5/3}$, where $k_{\bot } $ denotes the perpendicular wavenumber. This is the case that we consider for the feedback M-I coupling with nonlinearity. Indeed, a continuous power spectrum of the Alfvénic turbulence is found in the nonlinear regime of the feedback instability (Watanabe et al. 2016). It is qualitatively consistent with FAST observations of the power law spectrum of dispersive Alfvén waves (Chaston et al. 2008). More detailed theoretical studies and quantitative comparisons with observations are desired to determine what type of auroral structures are caused through turbulent Alfvénic wave interactions of the magnetosphere and the ionosphere.

Much of the previous analysis of the feedback instability has assumed a slab ionosphere described by height-integrated Pedersen and Hall conductances. However, the ionosphere has a finite thickness and an inhomogeneous profile of the conductivities and the Alfvén speed. An analysis of the feedback instability in a model including a full altitude profile of the Pedersen conductivity was conducted by Sydorenko and Rankin (2017). They noted that the inhomogeneity in the ion-neutral collision frequency produced a large shear in the Pedersen drift, and considered that this shear tends to smear out the density perturbations produced by the feedback interaction, preventing this interaction from going unstable. Later, Watanabe and Maeyama (2018) performed an eigenmode analysis of the instability in a model with an inhomogeneous ionosphere and found that unstable eigenmodes exists for cases with long parallel wavelengths in field line resonances, where the effect of inhomogeneity in the ionosphere is much less significant than in the IAR. Further work will be necessary to clarify under which conditions, if any, the feedback instability exists.

However, the feedback interaction may be effective at generating small-scale currents even in the absence of an instability. For example, Russell et al. (2013) performed numerical experiments in which the magnetosphere was assumed to be uniform, so that the reflection of waves from the ionospheric density gradient or the conjugate ionosphere, which is required for the feedback instability, was not present. Despite this, they found that in the downward current region, the upward motion of the electrons led to a depletion of the ionospheric density. This created conditions favorable to the small-scale structuring of the field-aligned currents. Sounding rocket results from Lynch et al. (2015) have shown that conductivity enhancements caused by the precipitation of electrons can also lead to structuring of the field-aligned currents. In addition, they point to the role of the neutral wind dynamo as an important effect in the structuring of currents. These results make it clear that the further investigations of the magnetosphere-ionosphere interaction are required to understand the structuring of auroral currents.

### Ionospheric Alfvén Resonator

In the ionosphere, a resonant cavity known as the Ionospheric Alfvén Resonator (IAR) can form when the plasma density drops more steeply with altitude than the decrease in strength of the dipole magnetic field. The IAR has an Alfvén speed profile that increases to a peak value in the range 4000-8000 km before dropping more slowly at higher altitudes. It was first identified by Polyakov and Rapoport (1981) during ionospheric heating experiments. Subsequently, Trakhtengertz and Feldstein (1984, 1991), and Lysak (1991, 1993), discussed the potential importance of the IAR in the context of auroral region physics. They showed that the sharp gradient in the Alfvén speed in the IAR results in partial reflection of Alfvén waves, trapping a fraction of their energy between the bottom of the ionosphere and the Alfvén speed peak. As will be shown below, the IAR is associated with electron acceleration on spatial and temporal scales characteristic of small-scale, dynamic aurora. For typical ionospheric parameters, the fundamental IAR resonance frequency is in the 0.1-1.0 Hz range.

A comprehensive theory of the IAR was derived by Lysak and Yoshikawa (2006), who suggested the trapped resonant wave modes could perhaps explain observations of broadband precipitating electrons with energies ranging from 100 eV to over one keV. The particle distributions of these precipitating electrons were identified as superthermal electron bursts (e.g., Johnstone and Winningham 1982; McFadden et al. 1986, 1987); however, definitive identification of the association between these types of bursts and Alfvén waves in the IAR came from NASA FAST satellite observations (e.g., Chaston et al. 2000, 2002a,b, 2003a,b). In a series of publications, Chaston demonstrated that the Alfvénic aurora is consistent with the particle distributions measured by FAST. The evidence lies in the fact that a broadband wave distribution means that electrons see a fluctuating parallel electric field during their passage through the acceleration region. An electron of energy 100 eV travels at roughly 1 R_E_ s^−1^, and thus for an acceleration length of around 1 R_E_, fluctuations on time scales of seconds are required to produce the observed particle distributions. Test particle modeling established this process is possible (Thompson and Lysak 1996), while later more detailed test particle simulations compare well with FAST data (Chaston et al. 2002a,b), confirming the Alfvénic nature of the acceleration process observed by FAST.

The focus here is on developments since the previous ISSI book on the aurora (Paschmann et al. 2003). Models of the IAR developed before 2003 generally considered the linearized MHD equations, with the addition of two-fluid effects of finite electron inertia and electron thermal pressure. Later, these two-dimensional models were extended to include the nonlinear Lorentz force due to field-aligned currents on auroral flux tubes (Sydorenko et al. 2008; Streltsov and Lotko 2008). These authors considered a geometry in which the $z$-direction is along geomagnetic field lines, the $y$-direction is azimuthal, and the $x$-direction completes the right-hand system. Since Alfvén waves have an electric field in the $x$-direction, and a magnetic perturbation in the $y$-direction, this leads to a nonlinear ponderomotive force, $j_{x} B_{y}$ directed upward along the geomagnetic field. Sydorenko et al. (2008) and Streltsov and Lotko (2008) described how this force accelerates ions away from the acceleration region to form a density cavity that enhances the ability of the plasma to support a parallel electric field. The generation of parallel electrostatic fields together with low density cavities is a positive feedback process in auroral acceleration regions, since the increased parallel electric field also contributes to the acceleration of ions and electrons out of the acceleration region, deepening the density cavity (Song and Lysak 2006). This process is a result of Alfvénic interactions in the solar wind-magnetosphere-ionosphere coupling system.

Phase mixing at the boundaries of these density cavities leads to a decrease in the perpendicular wavelength, further enhancing the parallel electric field, as described by Lysak and Song (2008), who prescribed a density cavity rather than having it form self-consistently. The structuring of waves perpendicular to the geomagnetic field was confirmed using observations from the FAST satellite (Chaston et al. 2006), which showed that density depletion was associated with ion outflow produced by Alfvén waves. Similar physics operates in field line resonances, where low frequency standing waves produce density cavities, perpendicular structuring and enhancement of parallel electric fields, and ion outflow (Rankin et al. 1999a,b; Lu et al. 2003a,b).

While early models of the IAR assumed that the field lines were vertical, measurements at low latitude (Simões et al. 2012) from the C/NOFS satellite have also shown IAR signatures. Lysak et al. (2013) developed a model in non-orthogonal dipole coordinates to investigate the development of Alfvén waves in the IAR at lower latitudes. This work has shown the existence of the IAR when magnetic dip angle is less than 90 degrees, although not to the equatorial latitudes described by Simões et al. (2012).

The modeling work of Lysak et al. (2013) also confirmed earlier suggestions (e.g., Greifinger and Greifinger 1968; Fraser 1975a,b; Fujita and Tamao 1988; Knudsen et al. 1992; Neudegg et al. 1995) that in the presence of Hall conductivity, shear Alfvén waves can mode convert to the fast wave. While the shear Alfvén mode propagates along the field line, the fast mode can propagate across magnetic field lines, and so the sharp increase in the Alfvén speed leads to an ionospheric waveguide. Thus, the incident shear Alfvén wave can produce ground signatures 100’s of kilometers from the source. The process was modeled by Woodroffe and Lysak (2012) who showed that the polarization pattern from such a localized source is consistent with the observations (Fraser 1975b).

A great deal of work on the IAR in the last 15 years has focused on verifying the expected signatures of this structure with satellites and sounding rockets as well as ground observations. Much of this work has considered the ratio of the perpendicular electric field to the orthogonal component of the magnetic perturbation and their phase difference. For a static sheet-like current system, field-aligned currents are closed by the presence of Pedersen conductance, for which this ratio is

5 $$ \delta E_{x}/\delta B_{y} = 1/\mu _{0}\Sigma _{\mathrm{P}}. $$

This expression uses the same coordinates defined earlier. On the other hand, for an inertial Alfvén wave, this ratio is

6 $$ \delta E_{x}/\delta B_{y} = V_{A}[(1+k_{\bot }{}^{2}\lambda _{e}{}^{2})(1+ k_{\bot }{}^{2}\rho _{i}{}^{2}))^{1/2}. $$

Here, we have included the finite ion gyroradius term but have neglected the electron kinetic term (often written in terms of the ion acoustic gyroradius) since it is not usually important at low altitudes in the IAR. For brevity, we will refer to this ratio as the “E/B ratio” in the discussion that follows. For typical parameters, the ratio indicated by Eq. (6) is usually much less than that given by Eq. (5). For both of these cases, the phase shift between $\delta E_{x}$ and $\delta B_{y}$ is 0 or 180 degrees, depending on the direction of the geomagnetic field. On the other hand, the IAR is characterized by the interference of up and down going waves and so the phase shift for a strong gradient in Alfvén speed oscillates around ±90 degrees, switching over when the mode structure of the IAR has an approximate node in either the electric or magnetic field at the point of observation (e.g., Lysak 1991). In addition, the E/B ratio becomes a function of frequency, becoming quite large, for example, when the standing wave pattern has a node in the magnetic field at the point of observation. In general, for realistic (Chapman-layer-like) ionospheric density profiles the magnitude of the reflection coefficient for Alfvén waves at the lower boundary of the IAR is less than unity, and often much less, leading to $E-\delta B$ phase shifts much smaller than ±90 degrees, as shown by Knudsen et al. (1992), who verified these predictions with sounding rocket observations. Furthermore, the ionospheric reflection coefficient varies strongly with frequency in the same range as the characteristic frequencies of the IAR (of the order of 1 Hz). This is due to the decrease of the effective conductivity due to the collisional skin depth effect, which becomes smaller than the ionospheric thickness at these frequencies (Lysak et al. 2013), as well as coupling to the fast mode.

Using Akebono data, Hirano et al. (2005) investigated the properties of waves in the IAR at three different altitudes, roughly 2000 km, 4000 km and 7500 km. They showed that at the lowest altitude range the E/B ratio is lower, with E/B approaching $1/\mu_{0}\Sigma_{\mathrm{P}}$, while at higher altitudes they were more consistent with E/B = $\text{V}_{\mathrm{A}}$. In addition, they showed that the phase relationship between E and B was consistent with the interference of waves in the IAR. One interesting observation is that the ratio at 2000 km altitude approached the inertial Alfvén wave case discussed above for perpendicular wavelengths shorter than 1 km. This may be because these short wavelength waves can be absorbed in the ionosphere (e.g., Forget et al. 1991; Lessard and Knudsen 2001).

Similar observations have been carried out more recently using the enhanced Polar Outflow Probe (ePOP) and the Swarm constellation of satellites (Miles et al. 2018). They observed that the E/B ratio approached the ionospheric values for lower frequency, consistent with the interpretation that the ionospheric E/B ratio should hold within a fraction of one wavelength of the ionosphere, which would be a larger distance for low frequency waves. Pakhotin et al. (2018, 2020) used the multi-point measurements from Swarm to verify the time-dependent nature of the wave structures, and also considered the field ratio and phase difference as a function of frequency to indicate correspondence with the standing wave model. Moreover, they pointed out that the low-frequency E/B ratio could be used as an independent measure of the Pedersen conductance in the ionosphere.

Wu et al. (2020a) carried out a statistical study of regions of intense $E$ and $\delta B$ field fluctuations in the 0-8 Hz range, selecting for enhanced correlations between the two fields as an indication of Alfvénic activity. Those authors searched for a consistent pattern between Alfvénic fluctuations, field-aligned currents, and auroral arcs observed with white-light all-sky cameras, and found no one-to-one relation between them. Rather, regions of enhanced Alfvén waves occurred within both upward and downward field-aligned current regions, and at their boundaries, and at varying distances away from bright auroral arcs. One conclusion that can be drawn from that study is that the ultimate sources of Alfvénic (and IAR) energy remain to be identified.

This combination of new model developments and more detailed observations have firmly established that the ionospheric Alfvén resonator is an important structure for the development of auroral field-aligned currents and the resulting particle acceleration. The IAR is particularly important in the evolution of small-scale structures when coupling with the ionospheric feedback interactions discussed in the previous section of this publication.

### Conclusions

In recent decades, our understanding of small-scale dynamic auroras has been rapidly advanced via both improved observations and theories. Advancements in camera technology have been parallelled by developments of in situ plasma measurements, which demonstrate magnetic field-aligned electron beams that appear bursty in the spacecraft frame during active periods such as auroral breakup. However, such measurements are often challenging to interpret given the space-time ambiguity. A suborbital sounding rocket crosses a 1-km-wide structure in 1 s, which is also the characteristic lifetime of such a structure as observed by ground-based cameras. Thus it is difficult to simultaneously resolve event lifetimes and spatial scales, both of which are needed to constrain auroral theories. Camera imaging can be used to resolve this ambiguity when available; another approach is the use of multiple spacecraft. More complete specification of the dynamic structure of small-scale aurora in space and time remains an important research goal. New development will also be valuable in carefully resolving the vertical luminosity distribution of small-scale dynamic auroras (Ivchenko et al. 2005; Dahlgren et al. 2013; Tuttle et al. 2014; Kataoka et al. 2016), which is complementary information to that of the multi-spectral observations (Dahlgren et al. 2008; Kataoka et al. 2011a,b).

Future observations of the various types of dynamic auroral forms should be put into context if the community is to understand what causes them and what their impact on the system might be. Future reports of the occurrence of these forms should state, as appropriate, (1) the geomagnetic activity level, (2) the phase of substorms, (3) the local time, (4) the relative latitude in the auroral zone (e.g. high-latitude or low-latitude), and (5) what other auroral forms are present and where is the dynamic form located relative to those other aurora.

On the theory side, Alfvén waves play an essential role in the formation of small-scale dynamic auroras. Several different cascading modes of Alfvén waves can form a variety of fine structures. For example, K-H and tearing instabilities of an arc lead to “folds” and “curls” (Hallinan and Davis 1970; Wagner et al. 1983; Wu and Seyler 2003; Chaston and Seki 2010), although reconnection in the M-I region also contributes to a particular type of vortical motions of auroras (Chaston 2015b). DAWs play the essential role for causing “packets” (Semeter et al. 2008) and “rays” (Lynch et al. 2012). The interference of resonant EMIC waves cause “flickering” (Temerin et al. 1986; Sakanoi et al. 2005). Consistent theories have been therefore proposed for small-scale dynamic auroras. However, predictive simulations have not been tested yet. In other words, it is still an open question how we can predict the occurrence of small-scale dynamic auroras such as curls, folds, flickering, and packets.

In future, 3-D kinetic simulations that extend from the equator to the ionosphere should be made to understand the driving of the Alfvénic perturbations and their dynamics in the presence of self-consistently formed parallel electric fields, based on the future investigations of the Alfvénic energy source.

What is the essential role of the filamentation of auroras for global energetics? Cross-scale coupling has been poorly investigated by observations. Continuous international effort is therefore needed for systematic monitoring observations of both global and local auroras at the same time, which would be necessary to build a firm empirical relationship among global-scale, mesoscale, and the small-scale dynamic auroras as reviewed in this article. Systematic surveys have also been conducted to identify the large-scale context of the small-scale structures, such as large-scale upward current or downward current (Liang et al. 2019; Wu et al. 2020a, 2020b). Next generation high-resolution global imaging of aurora from spacecraft would be a dream, but for the time being, rapidly increasing resolution of ground-based all-sky cameras, the networked distributions of imagery and small spacecraft, and timely data sharing combined with realistic simulations will renovate this aspect. Citizen scientists can play a key role in providing high quality opportunistic observations, as they have in the study of STEVE (MacDonald et al. 2018) and its mysterious filamentary structures (Semeter et al. 2020). Quantitative measurement of the filamentary structures using ground-based facilities combined with cutting-edge cameras would be essential to accumulate the quantitative knowledge of small-scale dynamic auroras (Lanchester et al. 1994, 1997, 1998, 2001), which has been taken over to the ongoing EISCAT 3D project.

## Supplementary Information

Below are the links to the electronic supplementary material.

Typical example of auroral arcs associated with an inverted-V type electron precipitation obtained at N2 1NG (428 nm), OI (558 nm), and N1 1PG (670 nm) by Reimei from 09:11:02 to 09:12:31 UT on 2005-12-26. A field-of-view of each channel is 7.6° × 7.6° which corresponds to an area of ∼70 km × 70 km at auroral emission height. Time and spatial resolutions are 120 ms and 1.1 km × 1.1 km, respectively. MLT is ∼0.8 and invariant latitude is from 74.0° to 73.1°. Rectangular dots indicate the location of magnetic footprint of the satellite (MPG 17.7 MB)

Example of multiple auroral arcs with shear motions obtained by Reimei from 10:06:04 10:06:20 UT on 2005-01-03. The format is the same as Movie 1. MLT is ∼0.5 and invariant latitude is from 72.3° to 71.3° (Asamura et al. 2009) (MP4 1.5 MB)

Example of flickering aurora in the brightest auroral form: Time: 2019-09-29/22:14 UT, Site: Tjornes, Iceland (66.2N, 17.0W, Invariant lat. = 65.9 °, MLT = UT-0.5) FOV: 1.9° × 7.6° (3.3 km × 13.2 km @100 km alt.), directed to magnetic zenith, Image size: 256 × 64 pixel; Sampling rate: 200 fps (MP4 3.1 MB)

Example of flickering aurora. Real-time play from 2006-10-22/18:16 UT. Site: Tromsø, Norway (69.6N, 19.2E, Invariant lat. = 66.6°, MLT = UT + 2.1); FOV 3.1° × 3.1° (5.4 km × 5.4 km @ 100 km alt.), directed to magnetic zenith; Image size: 256 × 256 pixel; Sampling rate: 32 fps; Filter: N2 1P 673.0 nm (AVI 11.6 MB)

Example of curls. The curl structure appeared from 0.0 s to 0.5 s from 2019-09-29/22:14:45 UT. The format is the same as Movie 3 (MP4 3.9 MB)

Example of a curl. Real-time play from 2008-01-09/12:25 UT. Site: Longyearbyen, Svalbard, Norway (78.2N, 16.0E, Invariant lat. = 75.6°, MLT = UT + 2.6); FOV 3.1° × 3.1° (5.4 km × 5.4 km @ 100 km alt.), directed to magnetic zenith; Image size: 256 × 256 pixel; Sampling rate: 20 fps; Filter: N2 1P 673.0 nm (AVI 3.0 MB)

Example of ruffs. Real-time play from 2006-11-23/21:38 UT. Site: Tromsø, Norway (69.6N, 19.2E, Invariant lat. = 66.6°, MLT = UT + 2.1); FOV 3.1° × 3.1° (5.4 km × 5.4 km @ 100 km alt.), directed to magnetic zenith; Image size: 256 × 256 pixel; Sampling rate: 20 fps; Filter: O^2+^ 1N 562.0 nm (AVI 9.3 MB)

Example of folds: Real-time play of 10-s time variation from 04:25:00 UT to 04:25:10 UT on 19 February 2014. The field-of-view is 15 deg by 15 deg, 100 fps, magnetic zenith is about the image center, north is to the top, and west is to the right (MPEG 27.0 MB)

Example of side-view rays appeared in PBI aurora: Time: 2019-10-01/23:36 UT, Site: Tjornes, Iceland (66.2 N, 17.0 W, Invariant lat. = 65.9°, MLT = UT − 0.5), FOV: 11.4° × 5.7°, directed to ∼30° west from magnetic north at an elevation angle of ∼10°, Image size: 384 × 192 pixel, Sampling rate: 250 fps (MP4 676 kB)

Optical emissions produced by the High-frequency Active Auroral Research Program (HAARP) ionospheric heating facility in Alaska. The emissions, recorded using an oxygen 777.4-nm filter, resemble field-aligned filamentary structure observed in natural aurora (Kendall et al. 2010) (MP4 642 kB)

Example of packets. Zenith-view EMCCD camera (9 deg × 9 deg field of view, 0.033 s exposure time) installed at Poker Flat Research Range (65.13N, 147.47W) (After Semeter et al. 2008) (MP4 1.0 MB)
